# Rice Chalky Ring Formation Caused by Temporal Reduction in Starch Biosynthesis during Osmotic Adjustment under Foehn-Induced Dry Wind

**DOI:** 10.1371/journal.pone.0110374

**Published:** 2014-10-20

**Authors:** Hiroshi Wada, Chisato Masumoto-Kubo, Yousef Gholipour, Hiroshi Nonami, Fukuyo Tanaka, Rosa Erra-Balsells, Koichi Tsutsumi, Kenzo Hiraoka, Satoshi Morita

**Affiliations:** 1 Kyushu Okinawa Agricultural Research Center, National Agriculture and Food Research Organization, Chikugo, Fukuoka, Japan; 2 Department of Biomechanical Systems, Faculty of Agriculture, Ehime University, Matsuyama, Ehime, Japan; 3 Agricultural Research Center, National Agriculture and Food Research Organization, Tsukuba, Ibaraki, Japan; 4 Department of Organic Chemistry-CIHIDECAR, Faculty of Exact and Natural Sciences, University of Buenos Aires, Buenos Aires, Argentina; 5 Clean Energy Research Center, The University of Yamanashi, Kofu, Yamanashi, Japan; Institute of Botany, Chinese Academy of Sciences, China

## Abstract

Foehn-like extreme hot and dry wind conditions (34°C, >2.5 kPa vapor pressure deficit, and 7 m s^−1^) strongly affect grain quality in rice (*Oryza sativa* L.). This is a current concern because of the increasing frequency and intensity of combined heat and water-deficit stress under climate change. Foehn-induced dry wind conditions during the grain-filling stage increase ring-shaped chalkiness as a result of spatiotemporal reduction in starch accumulation in the endosperm, but kernel growth is sometimes maintained by osmotic adjustment. Here, we assess the effects of dry wind on chalky ring formation in environmentally controlled growth chambers. Our results showed that hot and dry wind conditions that lasted for >24 h dramatically increased chalky ring formation. Hot and dry wind conditions temporarily reduced panicle water potential to –0.65 MPa; however, kernel growth was maintained by osmotic adjustment at control levels with increased transport of assimilate to the growing kernels. Dynamic tracer analysis with a nano-electrospray-ionization Orbitrap mass spectrometer and quantitative polymerase chain reaction analysis revealed that starch degradation was negligible in the short-term treatment. Overall expression of starch synthesis-related genes was found to be down-regulated at moderately low water potential. Because the events observed at low water potential preceded the packing of starch granules in cells, we concluded that reduced rates of starch biosynthesis play a central role in the events of cellular metabolism that are altered at osmotic adjustment, which leads to chalky ring formation under short-term hot and dry wind conditions.

## Introduction

It has been recognized that foehn-like high-speed hot and dry wind (e.g., 34°C, >2.5 kPa vapor pressure deficit [VPD], and 7 m s^−1^) disturbs the quality of rice (*Oryza sativa* L.) grain appearance [1], [2]. As the frequency and intensity of dryness are likely to increase in eastern Asia in addition to elevated global temperature under climate change [3], understanding the mechanism(s) behind rice quality under the combined stressors of heat and water deficit has become increasingly important in rice production. Hot and dry wind conditions during grain filling often impose temporary water deficit in plant shoots as a result of increasing VPD at elevated temperatures, resulting in a significant increase in ring-shaped chalky kernels, called ‘milky white rice’ (MWR) [4]. These kernels exhibit an annual ring-like chalky area on their transverse section that is typically composed of several cell layers in the endosperm, in which inadequate starch accumulation occurs at the midway of starch accumulation that occurs from the center towards outward in the endosperms [5], [6]. Loosely packed starch granules are observed in the interior of the cells, and air spaces between starch granules [7], [8] cause random light reflection [8], [9] to create the appearance of a chalky ring in the endosperm.

An interesting body of evidence has emerged in recent years shows that the regulation of several *α*-amylase-encoded genes plays a central role in chalky ring formation under long-term high-temperature conditions [10], [11], [12]. Because the chalky zone occurs midway between the center and peripheral part of the endosperm in the developing kernels, the ring-shaped chalkiness could be a cell-specific event associated with the disruption of starch accumulation under dry wind conditions [4]. Given that foehn-induced dry wind conditions are a result of the simultaneous occurrence of high temperature and water deficit (i.e., temporary shoot water deficit at elevated temperature) over a relatively short time frame, starch breakdown in kernels by *α*-amylase may occur similarly to that which has been reported under long-term high temperature [10], [12]. While it remains unknown whether starch breakdown or a reduction in starch synthesis occurs during induction of chalky formation under dry wind conditions, direct examination of the possibility of starch breakdown at the metabolic level is methodologically challenging.

In the last decade, metabolomic approaches have been widely applied in many areas of biological research due to the newest introduction of the Orbitrap mass analyzer to the family of high-resolution high-sensitivity mass spectrometers [13]. The Orbitrap-based mass spectrometer (MS) is an innovative and powerful instrument with high mass accuracy that allows useful measurements, including the correct measurement of isotopic ratios, in many analytical applications [14]. To our knowledge, this analyzer has not yet been used to determine the ratio of the carbon stable isotope (^13^C) prefixed in plants; however, it is possible to examine the significance of starch degradation under short-term dry wind conditions by analyzing the enzymatic degradation of starch in kernels pre-fixed with ^13^C. Here, we combined a pre-fixed ^13^C tracer experiment with nano-electrospray ionization (nano-ESI)-Orbitrap MS to determine the isotopic ratio of ^13^C-labeled glucose from the surface of starch granules newly synthesized in developing kernels, according to the partial starch degradation method [15].

In our previous study, we applied a dry wind treatment following a low radiation treatment to replicate foehn-associated climate conditions observed in the field [4]. Here, we sought to provide a better understanding of the mechanisms of chalky ring formation, which has often been recorded in field conditions, in developing endosperm under repeated hot and dry wind conditions (34°C, >2.5 kPa VPD, and 7 m s^−1^) in an environmentally controlled growth chamber for 24–72 h (representing potential foehn duration). In addition to the pre-fixed ^13^C isotope tracer experiment at the whole-plant level, plant water-status measurements–including an in situ endosperm turgor (Ψ_p_) assay–were conducted under 24-h dry wind conditions. Quantitative polymerase chain reaction (qPCR) analysis was also carried out for the target genes, including starch metabolism-related genes, to determine the impact of starch breakdown and altered starch synthesis on dry wind-induced chalky ring formation.

## Materials and Methods

### Plant Materials

**Figure 1:**
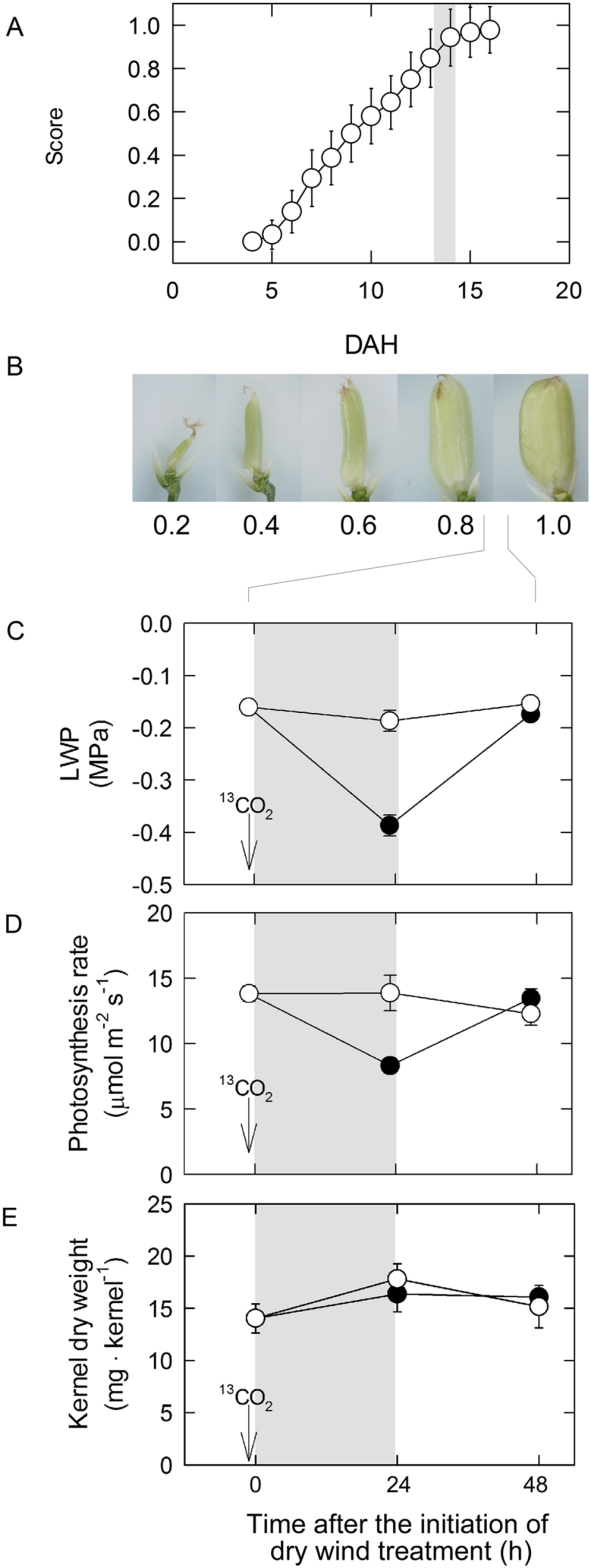
Time course of changes in kernel growth score (A) visually observed through hull; definition of kernel growth scores, 0 through 1.0 (B); and changes in leaf water potential (LWP) (C), photosynthesis rate (D), and kernel weight (E) under 24 h dry wind conditions, starting at 13 days after heading (DAH). Open and closed circles indicate control and dry wind treatments, respectively. Gray area indicates the 24-h dry wind treatment. For A, each point is the mean ± SD of at least 20 kernels from 4 different plants; for C–E, each point is the mean ± SE of 3–6 samples from different plants.

A growth-chamber experiment was conducted in 2011 and 2012 according to the previous study [4]. Simply, *Oryza sativa* L. cv. Koshihikari plants were grown outdoors in pots until the flowering stage. Ten plants per pot were prepared; the tillers were periodically removed to restrict each plant to its main culm to minimize panicle-to-panicle variations. At 5 days after heading (DAH), the plants were transferred to a growth chamber (22/22°C, 70/80% relative humidity [RH], 0.79/0.53 kPa VPD, and 750 µmol photons m^−2 ^s^−1^ photosynthetically active radiation [PAR]) set at the plant canopy with a photoperiod of 14 h day/10 h night. At 13 DAH, when the score of inferior kernels attached to the tertiary pedicels on the fourth to sixth secondary rachis branches (middle panicle position) reached 0.87 on average (Fig. 1A), the plants were transferred to another growth chamber (34/34°C, 50/40% RH, 2.66/3.19 kPa VPD, and 750 µmol m^−2 ^s^−1^ PAR) to conduct 24, 48, and 72 h dry wind treatments (referred to as ‘24 h W’, ‘48 h W’, and ‘72 h W’, respectively), starting from 1200 h. The grain score ranges from 0 to 1 according to size and developmental stage, as shown in Figure 1A and 1B. Wind speed was set at approximately 7 m s^−1^ at the plant canopy. Other potted plants were kept in the same chamber in a cool and non-dry wind (control) treatment. Wind speed at the canopy in the control treatment was 0.2 m s^−1^. After the dry wind was stopped at 1200 h each day, plants were transferred to the control chamber to grow until 33 DAH, after which the plants were placed outdoors until 40 DAH (maturing stage). Plants were supplied with water daily. For all of the following analyses (in situ Ψ_p_, ^13^C tracer, and qPCR assays), inferior spikelets attached to the tertiary pedicels on the middle panicle position were used because they showed the highest frequency of ring-shaped chalkiness under dry wind conditions at that stage of development [4].

### Plant Water Status and Photosynthesis

All plant water status and photosynthesis measurements were conducted as described previously [4]. Briefly, panicle water potential (PWP), stem water potential (SWP), and leaf water potential (LWP) were determined using the pressure-chamber technique [16]. After determining PWP, the cell pressure probe technique [17] was used for a continuous in situ assay of Ψ_p_ of individual cells in the growing inner endosperm, from 370 to 810 µm below the epidermis [4]. The tips of glass microcapillaries were ground with a grinder to obtain a tip inner diameter between 2 and 4 µm. The Ψ_p_ of the endosperm cell layers, where starch granules tended to be loosely packed, was measured and the stability of Ψ_p_ over time was assessed according to Wada et al. [18]. In some cases, the kernel water potential of spikelets attached at the same position in a panicle was determined at 13 and 14 DAH with isopiestic thermocouple psychrometers [19] after determination of PWP. Net photosynthesis was measured concurrently with LWP using a Li-Cor LI-6400 photosynthesis meter (Li-Cor Inc., Lincoln, Nebraska, USA) between 1000 and 1200 h during the experiment.

### Pre-fixed ^13^C Isotope Tracer and Whole-Plant ^13^C Distribution Analyses

The plants were divided into two groups for the ^13^C feeding experiment to obtain two different treatments. The first group was labeled with ^13^CO_2_ at 12 DAH (one day before initiation of the dry wind treatment) for the following pre-fixed ^13^C analysis, and the second group was labeled at 13 DAH (just prior to application of the dry wind; arrows in Figure 1C–D) for the ^13^C distribution analysis. The flag leaf in both groups was exposed to ^13^CO_2_. ^13^CO_2_ was applied by gently enclosing the leaf inside a 1.27-L polyester gas sampling bag (45 µm thickness, Analytic-Barrier, Ohmi Odor Air Services Inc., Tokyo, Japan) with a plastic container containing 0.5 g Ba ^13^CO_3_ (99 atom % ^13^C) and ^13^CO_2_ was then generated by injecting 2 mL lactic acid from the outside of the bag. The leaf was allowed to assimilate ^13^C under the light conditions provided in the growth chamber for 20 min between 1130 and 1150 h.

After ^13^C feeding was completed in the first group, the flag leaf was instantly removed from the plants with a razor blade at the junction between leaf blade and leaf sheath to minimize further import of residual ^13^C from the leaf to the kernels, and the cut surface was immediately coated with Vaseline to avoid dehydration. The pre-fixed inferior kernels, attached to a similar position as used for the in situ Ψ_p_ assay, were harvested 24 h after the dry wind treatment for the following ^13^C isotope analysis. Extraction of the starch fraction prior to ^13^C isotope analysis was conducted using a modification of the method of Gholipour et al. [15]. Freeze-dried kernels, in a similar position to those used for the in situ Ψ_p_ assay, were homogenized with water/ethanol (1 4 v/v). After centrifuging for 20 min at 3000×*g*, starch in insoluble residue was washed twice with 50 mM sodium acetate buffer (pH 4.5), and solubilized starch was hydrolyzed to Glc by adding 100 L α-amyloglucosidase (2 mg mL^−1^) dissolved in sodium acetate buffer to 4 mL of starch solution and kept in a water bath at 55°C for 1 h. Sodiated Glc, which was the most abundantly yielded glucose ion including Glc (see Fig. S1 in File S1), was analyzed using a nano-ESI MS (Exactive Orbitrap LC-MS, Thermo Fisher Scientific Inc.), and the isotopic ratio of *m/z* = 204 (C_5_^13^CH_12_O_6_+^23^Na; Mm = 204.0561693) to *m/z* = 203 (C_6_H_12_O_6_+^23^Na; Mm = 203.0527693) was determined.

In the second group, plant parts were similarly harvested and separated into six components: flag leaf blade, flag leaf sheath, uppermost internode, whole panicle, inferior kernels, and other organs. Because the preliminary test and reference [20] both indicated little to no ^13^C distribution in roots at this stage, and because this estimate would not affect the analysis, roots were discarded to facilitate rapid completion of the dissection. The five tissue components were freeze-dried and the other organs were oven-dried with forced air, after which they were ground to a fine powder. For each tissue, approximately 1.0 mg well-mixed powder was used to determine total carbon and the isotopic ratio of ^12^C:^13^C using an element analyzer/isotopic ratio MS (ANCA-SL, SerCon Company, Cheshire, UK). Whole-plant ^13^C abundance was estimated according to Mohapatra et al. [20].

### Gene Expression Analysis

Total RNA was extracted from 13–20 mg fresh weight of four kernels per panicle using RNAs-ichi!-S (Rizo Inc., Tsukuba, Japan). The isolated RNA (4 µg) was treated with TURBO DNase (Life Technologies), and then reverse-transcribed using an iScript Advanced cDNA Synthesis kit (Bio-Rad) in a total volume of 20 µL. Quantitative real-time PCR was carried out in a real-time PCR system (CFX 96 Connect, Bio-Rad). Each reaction (5 µL) contained 300 nM of each primer, 0.5 µL of 1∶100 diluted cDNA, and 2.5 µL of SsoAdvanced SYBR Green Supermix (Bio-Rad). The thermal cycling conditions were 95°C for 30 s followed by 95°C for 5 s and 60°C for 10 s for 40 cycles. For amplification of *-amylase 1A, 2A*, *3A*, *3D*, and *invertase 3,* cDNA (1∶10 dilution) was used with an annealing/extension time of 30 s. Dissociation curves for each amplicon were then analyzed to verify the specificity of each amplification reaction; the dissociation curve was obtained by heating the amplicon from 65 to 95°C. No evidence of any primer dimer or formation of other non-specific product was detected for any of the primer pairs used. Each PCR was run in duplicate within the same plate, and the Ct values obtained from the technical replicates were averaged. The transcripts of 27 genes known from the RiceXPro gene expression database (http://ricexpro.dna.affrc.go.jp/) to exhibit strong expression in developing rice endosperm [21] were quantified by comparing the cycle threshold (CT) of the target gene with that of eukaryotic elongation factor-1α [22] and ubiquitin-conjugating enzyme E2 [23]. Gene expression was expressed as the mean and standard error of the three biological replicates. Primer sequences used for the amplification are listed in Table S2 in File S1.

### Kernel Quality and Weight

Spikelets in each portion in a panicle were further classified according to Matsuba [24]. The appearance of dehulled grains was objectively evaluated with a chalky rice grain predictor (RN-850, Kett Ltd., Tokyo, Japan) that is capable of determining the number of MWR kernels by analysis of scanned images of sliced kernels. Dry weight of kernel samples was determined during the treatment and at harvest [4].

### Microscopy

Transverse sections of MWR kernels collected at harvest were observed by scanning electron microscopy (SEM). Sections were placed on double-sided carbon tape and viewed under a field-emission SEM (Hitachi S-3500N; Hitachi Co., Hitachi, Japan) at an accelerating voltage of 8.0 kV. The SEM images taken of the chalky region were converted to 8-bit grayscale and binarized by using the intensity threshold function, and the spatial ratio of air space to total area (*k*) was determined using image software (Image J, NIH). Sections were also photographed at 50x using a digital microscope (KH-3000, Hirox, Tokyo, Japan). Using the images, the volume of kernel (*V^kernel^*) and chalky area (*V^chalk^*), not including the volume of inner translucent area, was individually calculated as an ellipsoid body according to the equation *V* = 4π*abc*/3, where *a*, *b*, and *c* denote the three semi-axis of an ellipsoid. The volumetric ratio (*l*) of air space observed in the chalky region in the kernel volume was estimated according to;
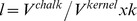
(1)


### Statistical Analysis

Analysis of all physiological data and rice appearance data was performed using Tukey’s Honestly Significant Difference (HSD) test, PROC ANOVA or Student’s *t*-test, PROC TTEST in SAS version 9.2 (SAS Institute, Cary, NC). A simple linear regression was performed between PWP and kernel water potential at 13 DAH.

## Results

### Rice Appearance

The rice appearance data showed that the proportion of ring-shaped chalky kernels, known as ‘milky-white rice,’ was increased substantially by application of dry wind treatment for 24 h, with a corresponding decrease in perfect rice (Table 1). There was no reduction in kernel weight under dry wind conditions up to 48 h, but ring-shaped chalkiness increased significantly (Table 1). A significant decline in final kernel weight was observed when the duration of dry wind extended to 72 h (Table 1). These results suggest that 24-h wind treatment represents a critical time point for initiating metabolic modifications related to the formation of chalkiness. Hence, we focused on the 24-h duration and conducted the subsequent carbon isotope and gene expression assays in the 24-h dry wind-treated kernels.

**Table 1:** Percentage of filled kernels, final kernel weight, and rice appearance of tertiary pedicels attached at the middle primary rachis branches, where an in situ turgor assay was conducted, in a panicle grown under 24, 48, or 72 h dry wind treatment in growth chambers.

Treatments	Filled kernels	Final kernelweight	Rice appearance^a^		
			PR	MWR	OR
	*%*	*Mg* · *kernel^−1^*	*%*	*%*	*%*
Control	90.2	23.0a^§^	95.0a	1.6c	3.5
24 h Wind	84.1	22.5ab	82.9ab	15.3bc	1.8
48 h Wind	84.2	21.8ab	69.0bc	29.4ab	1.6
72 h Wind	86.8	21.2b	56.2c	41.3a	2.5

a PR = perfect rice; MWR = milky white rice (ring-shaped chalky kernels); OR = other rice appearance.

Values in each treatment are means ± SE of four biological replications of panicles.

Means within a column followed by different letters are significantly different (*P*<0.05) (Tukey’s Honestly Significant Difference [HSD] test).

### Effects of Photosynthesis and Kernel Weight under Dry Wind Conditions

When plants were treated with dry wind for 24 h, LWP was significantly reduced but recovered within one day after stopping the wind treatment (Fig. 1C). Rates of photosynthesis declined to 60% that of the control treatment under shoot water deficit conditions, and became similar to that of the control on the day following the dry wind treatment (Fig. 1D). The average thickness of the boundary layer (measured as mean flag leaf length = 20 cm and numerical factor = 4.0) under control and dry wind conditions was 3.9 and 0.7 mm, respectively, according to Eq. (7.8) in Nobel [26], indicating that the boundary layer thickness was substantially reduced under the dry wind condition relative to the control. Kernel dry weight was similar between the control and dry wind treatments (Fig. 1E). There was also no significant difference (P<0.05) in fresh weight or water content between the treatments (data not shown). The dry weight of panicles collected at 24 h was 1.70±0.16 g and 1.70±0.15 g (mean ± SD, n = 6) for the control and dry wind treatment, respectively. There was no difference in spikelet numbers per panicle between the control and dry wind treatment (112±2 and 110±2 [mean ± SD, n = 6], respectively), indicating that there was no difference in sink size. These results indicate that rates of metabolite translocation to kernels were not affected by inhibition of photosynthesis in the short term.

### Water Relations of Plants and Endosperm

**Figure 2:**
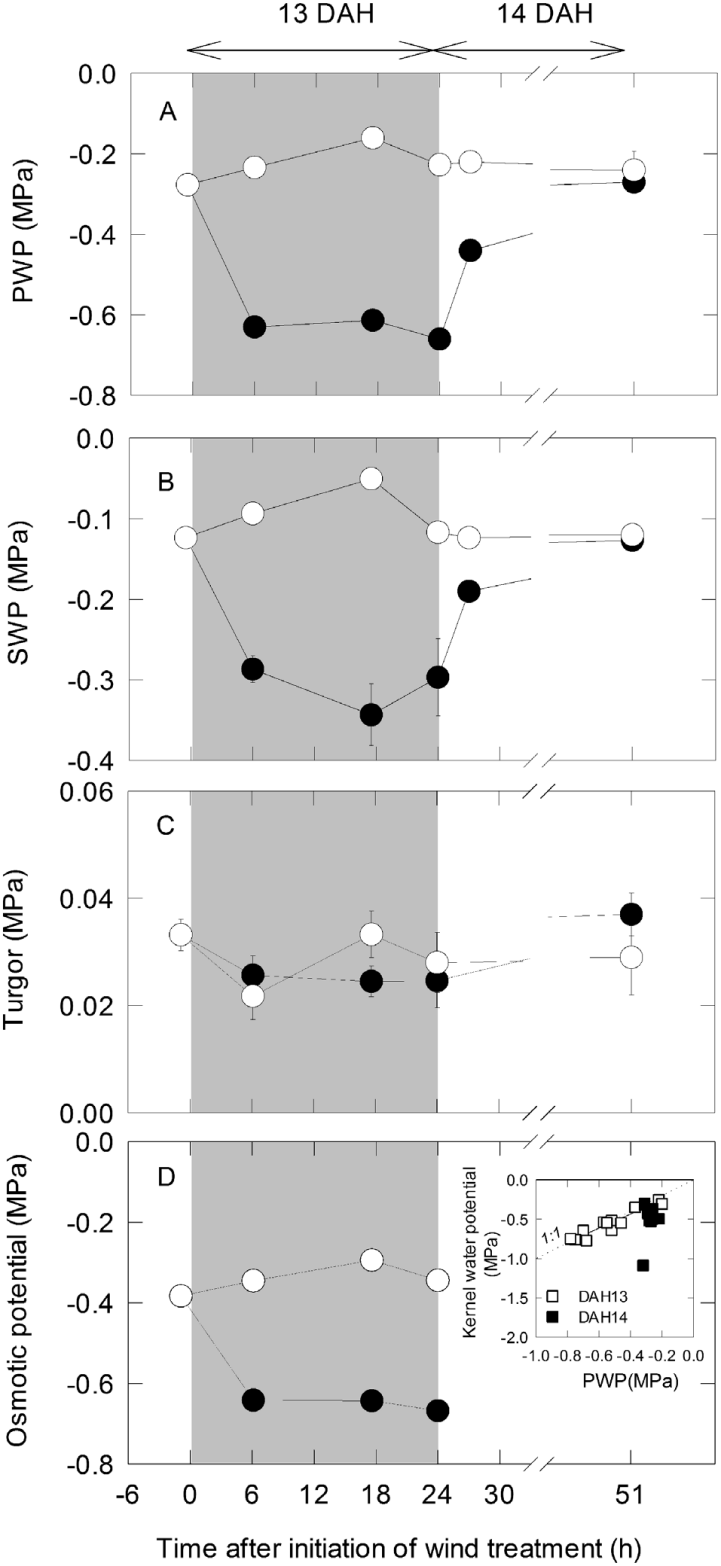
Changes in panicle water potential (PWP) (A), stem water potential (SWP) (B), endosperm cell turgor (C), and the weight-averaged calculated endosperm osmotic potential (D) in developing rice kernels under dry wind conditions. Open and closed circles indicate control and dry wind treatments, respectively. In D, the inset indicates kernel water potential measured with an isopiestic psychrometer as a function of panicle water potential measured with a pressure chamber in both the control and dry wind treatments, collected at 13 days after heading (DAH) (open squares) and 14 DAH (closed squares). The gray zone indicates the duration of dry wind treatment, corresponding to 13 DAH. In the inset in panel D, the slanted dotted line labeled ‘1∶1’ indicates an equipotential line. The solid line indicates the regression line at 13 DAH. For A and B, each point is the mean ± SE of 3–19 samples from different plants. For C, data are means ± SE of 4–22 cells measured in 2–8 inferior grains in the same location on a panicle.

After initiation of the dry wind treatment at 13 DAH, PWP decreased to approximately –0.6 MPa at 6 h, and remained at that value throughout the dry wind treatment (Fig. 2A). After the dry wind was stopped at 24 h, PWP began to increase and recovered to the level of the control on the following day (Fig. 2A). The SWP values were much higher than the PWP values, and SWP exhibited a similar pattern to PWP under dry wind conditions (Fig. 2B). Consequently, the gradient of water potential between stem and panicle under dry wind conditions was approximately 0.3 MPa greater than that of the control, and recovered to the level of the control within 24 h after stopping the treatment (data not shown). In the inner endosperm cells, Ψ_p_ was maintained overall, even at low PWP under dry wind conditions. The stability of Ψ_p_ over time was fairly high, with an average value of –0.001 MPa/min (*n* = 96). Maintenance of Ψ_p_ was observed until 51 h after initiation of the dry wind treatment (Fig. 2C). The tissue-averaged water potential of kernels collected at 13 DAH was highly correlated with PWP (*r* = 0.95, *P*<0.0001), which indicated that the relationship was linear and essentially equivalent across a broad range of PWP, ranging from –0.20 to –0.78 MPa in both treatments (inset in Fig. 2D). In contrast, there was no significant correlation (*r* = 0.26, *p* = 0.43) between the kernel water potential and PWP for the kernels collected on DAH14, presumably due to the lowering of water potential from the onset of kernel dehydration (inset in Fig. 2D). The osmotic potential of endosperm cells (Fig. 2D) was calculated by subtracting endosperm Ψ_p_ from kernel water potential, according to the correlation between kernel water potential and PWP observed at 13 DAH (inset in Fig. 2D). The osmotic potential of endosperm cells declined significantly as water deficit developed under dry wind conditions, reaching –0.65 MPa in 6 h and remaining at that value until the dry wind was stopped.

### No Significant Starch Degradation but Increased Assimilate Import to Kernels under Dry Wind Conditions

**Figure 3:**
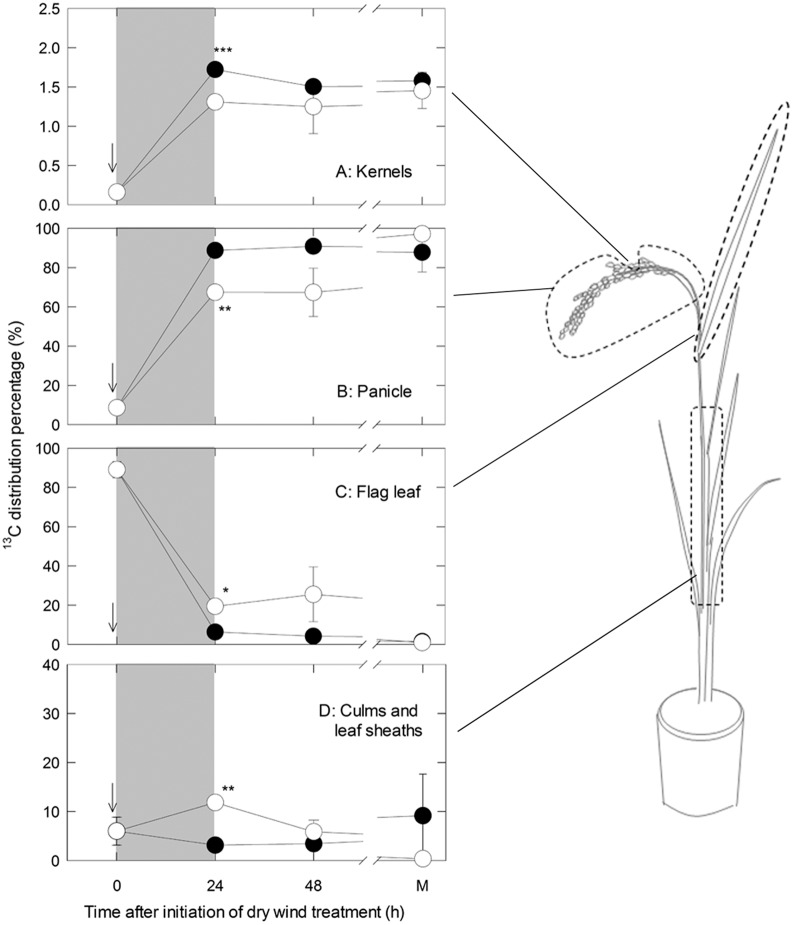
Time course of changes in ^13^C distribution in kernels located in the same position where an in situ turgor assay was conducted (A); in the panicle excluding the kernels (B); in the flag leaf (C); and in culms and leaf sheaths (determined as pooled organs with flag leaf sheath, uppermost internode, and other tissues) (D), corresponding to the sampled locations shown in the schematic potted plant to the right of the figure panels. ‘M’ on *x*-axis indicates maturation. The plants were labeled with ^13^CO_2_ for 20 min from the flag leaf at 13 DAH, just before the dry wind treatment was initiated, as indicated by the arrow. Open and closed circles indicate the control and dry wind treatments, respectively. Gray areas indicate the dry wind treatment. Each point is the mean ± SE of three samples from different plants. Significance at the 0.05, 0.001 and 0.0001 probability levels is indicated by *, **, and ***, respectively.

Plants were subjected to dry wind conditions after the carbon isotope was pre-fixed and synthesized as ^13^C-containing Glc on the surface of starch granules in the kernels. The mass spectrum from the starch fraction indicated that sodiated Glc was the most abundant (Fig. S1 in File S1). The isotopic ratio of sodiated C_5_^13^CH_12_O_6_ (*m/z* = 204) to sodiated C_6_H_12_O_6_ (*m/z* = 203) partially digested from starch extracted from the unfed kernels was 5.95%, whereas the isotopic ratio in the pre-fixed kernels just before applying the dry wind treatment (*T* = 0 h) was determined to be 6.58%. When the isotopic ratio was determined from the starch fraction collected at 24 h, there was no significant difference (*P* = 0.08) between the treatments (Table 2), with similar ^13^C distribution percentage in the kernels (data not shown). The isotopic ratio of the less-sodiated C_4_^13^C_2_H_12_O_6_ (*m/z* = 205) to sodiated C_6_H_12_O_6_ (*m/z* = 203) also indicated no difference between treatments (data not shown). Import of ^13^C to the kernels and panicle appeared to increase by approximately 1.3-fold relative to the control (Figs. 3A and B). Assimilate transport from the flag leaf was enhanced at 24 h after application of dry wind (Fig. 3C), and a temporal increase in assimilate supply from other organs (mostly culms and leaf sheaths) to sink tissues was observed (Fig. 3D). No significant difference between treatments was observed in any organs at maturation (Figs. 3A–D).

**Table 2:** Isotopic ratio of ^13^C-labeled sodiated glucose, (*m/z* = 204 to *m/z* = 203) detected in the ethanol-insoluble fraction in kernels sampled at 24 h after the initiation of dry wind treatment.

Treatments	Isotopic ratio
	*%*
Control	6.64±0.05
Wind	6.54±0.04
*P*-value	0.08

The plants were labeled with ^13^CO_2_ at 12 days after heading (DAH) from the flag leaf to pre-fix ^13^C in the kernels prior to initiation of the dry wind treatment at 13 DAH.

Values indicate the mean ± SE of three biological replications of two pooled kernels.

### Patterns of Gene Expression in Endosperm under Dry Wind

**Figure 4:**
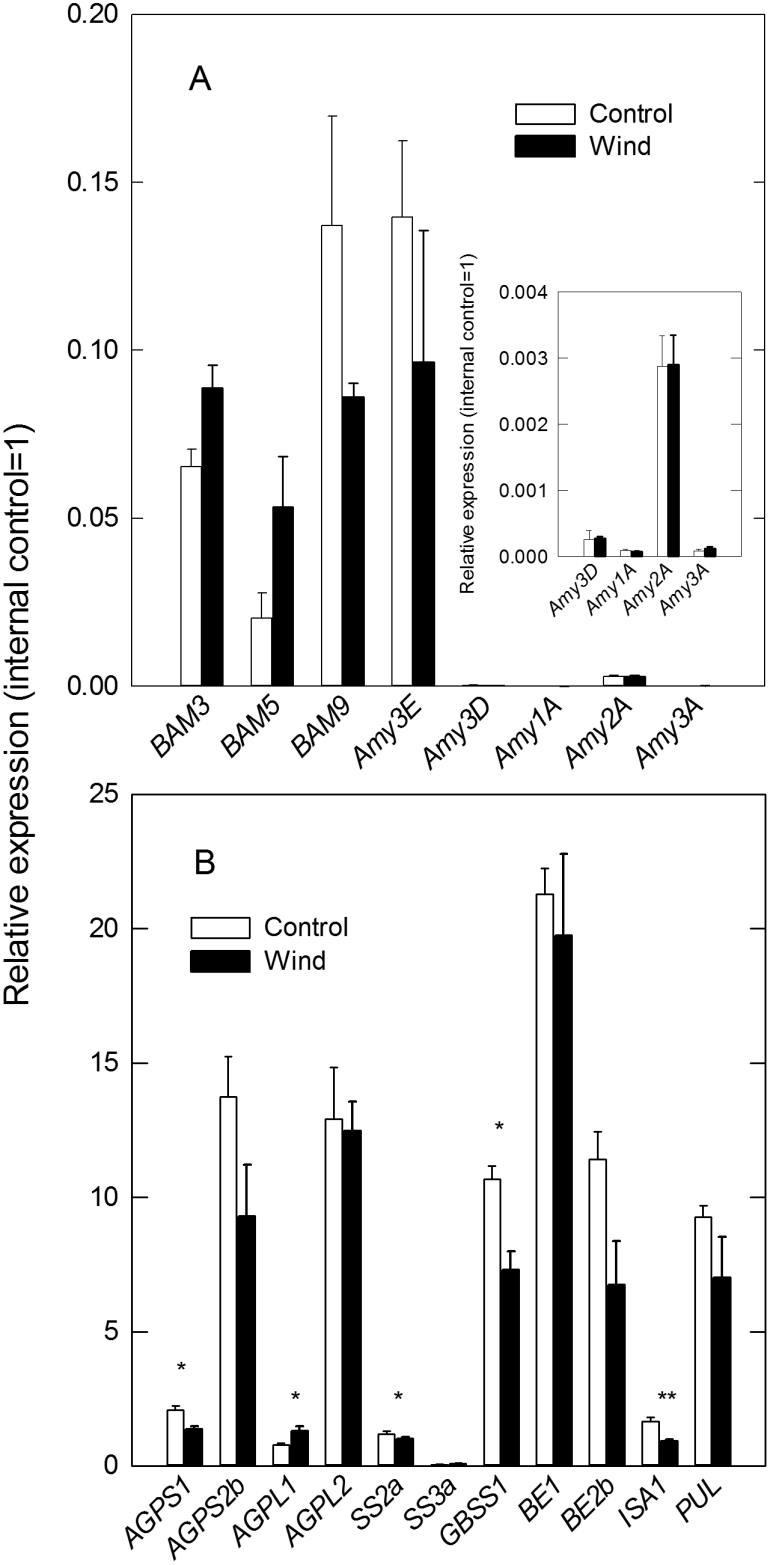
Expression profiling of starch degradation-related genes (A) and starch biosynthesis-related genes (B) in the growing kernels after 24 h of dry wind treatment. Inset in A is an expansion of the figure showing the expression of four α-amylase-encoding genes. Black and white bars indicate dry wind and control treatments, respectively. Data are the mean ± SE (*n* = 3) of four inferior kernels pooled at the position in the panicles where the in situ Ψ_p_ assay was conducted. Significance at the 0.05 and 0.01 probability levels is indicated by * and **, respectively.

To investigate the underlying molecular basis for chalky formation under dry wind condition, we analyzed 39 genes related to sugar and starch metabolism, hormone metabolism, and stress response. No significant difference was observed between the two treatments for any genes related to amylolytic degradation, including α-amylase and β-amylase-encoding genes (Fig. 4A). For starch biosynthesis, expression of ADP-glucose pyrophosphorylase large subunit 1-encoding gene (*AGPL1*, categorized as endosperm-preferred) [25], was up-regulated at a relatively low level, although expression of the rest of the starch metabolism-related genes was consistently down-regulated under dry wind conditions (Fig. 4B). In particular, the ratio of relative expression of the endosperm-specific genes *AGPS1* and *GBSS1* to the endosperm-preferred gene *ISA1* declined significantly under dry wind conditions compared to the control. Expression of invertase-encoding gene (*CIN2*), three sucrose synthase genes (*SuSy2*, *SuSy3*, and *SuSy4*), ADP-glucose transporter-encoding gene (*BT1-2*), and two sucrose transporter-encoding genes (*SUT1* and *SUT2*) was not significantly altered at 24 h, whereas expression of *cyPPDKB* was significantly reduced under dry wind conditions (Table S1 in File S1). In addition, small heat shock protein-encoding genes (*HSP16.9A*, *HSP17.9A*, *HSP18.0*, and *HSP26.7*), late embryogenesis-abundant protein-encoding genes (*LEA8* and *LEA21*), and ABA 8′-hydroxylase-encoding gene (*ABA8ox2*) were up-regulated, while expression of manganese superoxide dismutase 1 (*MSD1*) was significantly down-regulated (Table S1 in File S1).

### Kernel Anatomy and Starch Accumulation

**Figure 5:**
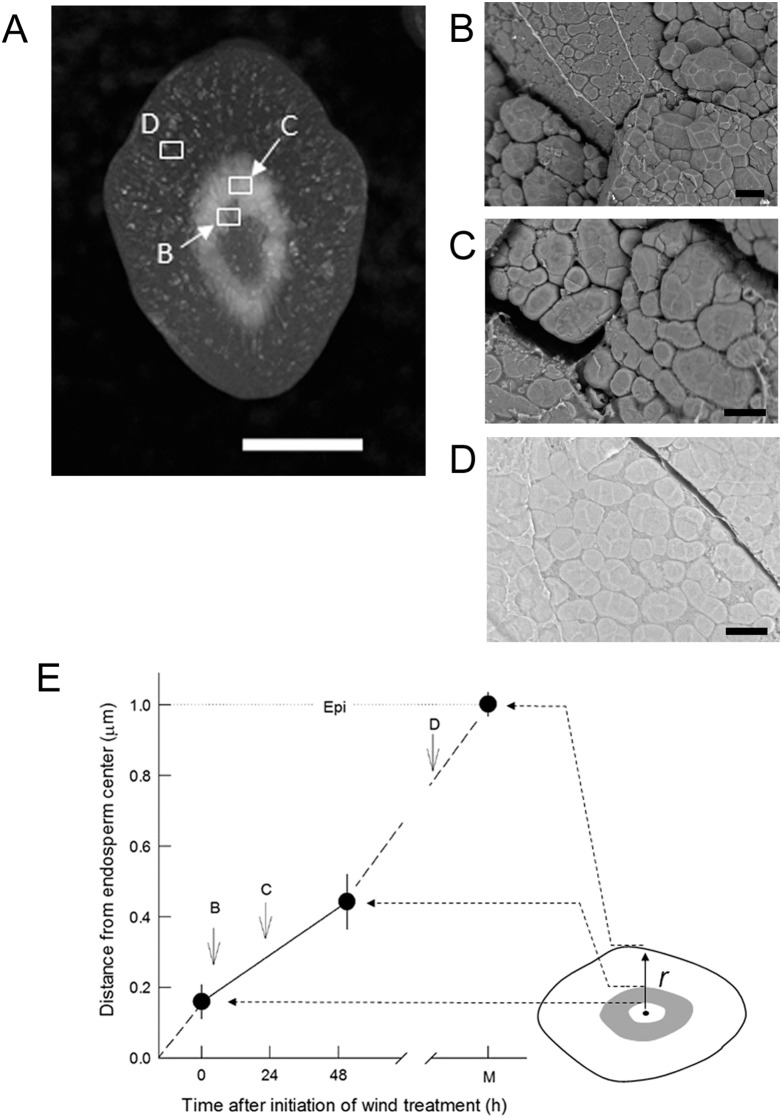
Transverse sections of a typical ring-shaped chalky kernel (A); and expanded SEM images taken at the three zones (B–D) shown in A. B, C, and D indicate the border between the inner translucent and chalky region (chalky cell in the left corner cell), the chalky region with loosely packed starch granules, and the translucent zone, respectively. The distance (*r*) from the endosperm center along the semi-minor axis in ring-shaped chalky kernels as a putative function of the duration of dry wind conditions (E). In E, the inner and outer borders of translucence and chalkiness correspond to the initiation of the dry wind treatment and the recovery point observed at 52 h in Fig. 2A, respectively; B, C, and D are indicated by arrows. ‘Epi’ indicates epidermis. In B–D, note that fissured fractures in each figure correspond to the cell wall. Bars in A and in B–D indicate 1 mm and 10 µm, respectively.

The transverse sections of ring-shaped chalky kernels indicated that inner endosperm cells with loosely packed starch granules typically formed over several cell layers. SEM observation clearly showed that the chalky region was sandwiched between two translucent areas (Fig. 5A). In the chalky region, heterogeneity in the size of amyloplasts caused the presence of air space in the cells (Figs. 5B and C). In contrast, SEM revealed densely packed starch granules in the translucent area, where the cytosol was well filled with the granules (Fig. 5D). The image analysis indicated that the spatial ratio of air space in the chalky cells, *k*, was 10.6±4.2% (mean ± SD, *n* = 11). Since the volumetric ratio of chalky region to kernel (*‘V^chalk^/V^kernel^*’, Eq. 1) was 13.3±3.5% (mean ± SD, *n* = 21), the average volumetric ratio of air space to kernel in the chalky region, *l*, was estimated as 1.41% using Eq. 1. In addition, no pits resulting from starch hydrolysis by amylolytic enzymes reported at high temperature (e.g., Reference [9]) were observed on the surface of starch granules in the chalky region when treated under short-term dry wind condition (Fig. 5C). It is noteworthy that an increase in air space in the chalky region did not contribute to weight loss in whole kernels (Table 1). This means that small metabolites accumulated in the symplast except for amyloplasts may not have been microscopically detected by SEM.

## Discussion

Our results indicate that hot and dry wind conditions during ripening, for the shortest duration known to have been recorded, induce chalky ring formation, which degrades rice appearance. We showed that a partial reduction in starch biosynthesis occurred concomitantly with osmotic adjustment and prior to chalky ring formation in endosperm cells under dry wind conditions. The combined analysis of dynamic pre-fixed ^13^C tracer (Table 2) and qPCR gene expression (Fig. 4, Table S2 in File S1) showed an overall reduction in starch biosynthesis and insignificant starch degradation in the short term. Kernel growth was maintained by osmotic adjustment under dry wind conditions, without a detectable reduction in kernel weight (Figs. 3 and 5), consistent with the previous work [4]. Given the similarity between the symptoms of progression of chalky ring formation and starch accumulation patterns in the developing rice endosperm [5], [6], it is very likely that the reduction in starch synthesis was temporary and recovered after the treatment was stopped, as the changes in water status occurred temporarily at high VPD during the dry wind treatment, as indicated in Figure 5E. This proposal is consistent with the suggestion about MWR provided earlier [27]. Hence, we propose that a temporary reduction in starch biosynthesis during osmotic adjustment is responsible for the ring-shaped chalkiness formed by dry wind-induced moderate water stress developed at high VPD, rather than an effect of high temperature, during the short term.

It has been observed that the occurrence of MWR increases under long-term (generally ≥4 days) high-temperature conditions (e.g., 4 and 15 days in References [28] and [10], respectively), although the duration of dry wind treatment (24–72 h) conducted here was much shorter than that of the high temperature treatment. Therefore, it is noteworthy that such a large occurrence of ring-shaped chalkiness (Table 1) was induced at low water potential under the high VPD environment, rather than by high temperature *per se*. While it has been also recognized that sink-source manipulation has a significant effect on the occurrence of MWR [30], no differences in sink size of plants were observed between treatments in this study. Therefore, other potential indirect effects on chalky rice formation related to the sink-source balance could be ruled out. In addition, there was no significant difference in kernel number and weight between the 24 h dry wind condition and the control, which indicates that kernel growth continued even at low water potential, resulting in no detectable final kernel weight loss (Fig. 1E) even with the increase in ring-shaped chalky kernels (Table 1). This observation is not surprising because the volumetric ratio of air space observed in the chalky region was negligible (≈1.4%) in ring-shaped chalky rice, and was insufficient to contribute to potential weight loss. In addition, small metabolites accumulated during osmotic adjustment must have additional extra weight.

The fact that a high absorption of ^13^C-labelled assimilates (Fig. 3) and active solute accumulation (Fig. 2D) were both observed at low water potential is strong evidence that the assimilate deficit *per se* does not account for the chalky ring formation under short-term dry wind conditions. Importantly, it was suggested that the onset of ring-shaped chalky formation was closely associated with the water status of the growing sink tissues (Fig. 2). Because the PWP observed here (approximately –0.65 MPa) was considerably higher than that often observed at spikelet desiccation (e.g., –1.3 MPa in reference [30]), the intensity of stress might have been fairly mild in this study. When the dry wind treatment was extended (see 72 h W in Table 1), MWR increased in number and kernel weight loss became significant, presumably as a result of insufficient assimilate delivery [31] and/or direct effects on starch biosynthesis (Fig. 4) in the kernels, as we observed here. The present study suggests another scenario that as water status declines and/or the duration of low water potential is extended, there might be a threshold above which starch metabolism is disturbed but kernel growth is ensured by increasing sugar delivery, at moderately low water potentials under dry wind conditions.

As in the previous work with low light intensity followed by dry wind treatments [4], here we have shown that Ψ_p_ is maintained by osmotic adjustment in endosperm cells under dry wind conditions regardless of the history of low light intensity. Moreover, this work showed that rates of starch biosynthesis were reduced by dry wind conditions. The observed dynamic changes in water status under dry wind conditions are likely to be closely associated with spatiotemporal metabolic modifications related to starch accumulation that occurred at the midway of the endosperm prior to ring-shaped chalky formation, as shown in Fig. 5E. Our data further showed that PWP was equivalent to the kernel water potential under high evaporative demand, at least before kernel desiccation occurred (inset in Fig. 2D). Since there was no detectable reduction in kernel weight (Table 1) with elevated assimilate delivery to the sink tissues (Fig. 3), even with the increase in chalkiness in moderately-stressed kernels, we interpret that kernel growth occurred at the control level as a consequence of Ψ_p_ maintenance (Fig. 2C) by osmotic adjustment while kernel thickness was increasing [4]. When the plants were temporally subjected to high-speed dry wind conditions and the water potential of air reached –98.3 MPa (at 34°C and 50% RH), cuticular transpiration should have increased at the spikelet surface that lacks stomatal control [32]. This would occur as a result of increased conductance of water vapor diffusing across the cuticular layers and increased xylem tension in the panicle according to the steep water potential gradient established between the air and panicle surface. Nonami and Hossain [33] showed that water fluxes for growth and transpiration were superimposed under high-VPD conditions. Based on our findings, it is likely that endosperm growth occurred by establishment of a growth-induced water potential gradient [34], [35] under foehn-induced high evaporative demand.

Under adverse climate conditions, such as low light intensity during ripening, a reduction in newly assimilated carbohydrates is compensated by enhanced translocation from non-structural carbohydrates pre-stored in culms and sheaths [36]. Likewise, an approximately 1.3-fold increase in ^13^C-labelled assimilates in the kernels from mature organs (Fig. 3) could have directly contributed to kernel development under the short-term dry wind conditions in spite of the 40% decline in rates of photosynthesis (Fig. 1D). Similar observations have been made for mature tissues at osmotic adjustment in other crops [37], [38]. An analysis of the data presented in the previous work [4] showed that there was a significant interaction between low light intensity and dry wind conditions on chalky ring formation, indicating that a history of low light intensity has an impact on chalky formation (data not shown). Low light intensity would also reduce the availability of assimilates stored prior to lowering of plant water status, and this assimilate supply determines kernel weight and quality, depending on the intensity and duration of the stress [39].

Molecular understanding of chalky formation at high temperatures during grain-filling has recently extended [10], [12], [40]. Although most work has concentrated on response to long-term high temperature, it is also important to understand how chalkiness induced by the combined stress of heat and water deficit, especially foehn-like short-term hot and dry wind conditions, would impact rice quality under accelerating global warming [3]. Foehn-induced dry wind conditions represent a simultaneous stress consisting of temporary shoot water deficit and high temperature, and therefore plant responses to these two different stressors might overlap. Yamakawa’s pioneering work showed that expression of several *α*-amylase-encoded genes, including *Amy1A* and *Amy3D*, was attributed to chalky formation at high temperature [10], [12]. However, this evidence has been called into question by Ishimaru et al. [41], who performed a spatial analysis of α-amylase-encoding genes in endosperm by using a laser micro-dissection method. These researchers seemed to observe different types of chalkiness (MWR with or without chalky ring formation). These inconsistencies might be explained by differences in the sampled sites, potentially depending on differences in the intensity and/or duration of stress.

In this work, we used the Orbitrap-based MS to determine the isotopic ratio of Glc from starch in combination with the partial enzymatic degradation [15] in the prefixed kernels. According to the calculated isotopic ratio of C_6_ molecules for carbon based on the ^13^C content (Reference [42]), the isotopic ratio should correspond to 6.50%. However, what we observed in the unfed kernels was determined to be 5.95%, lower than the theoretical value. The difference between theoretical and observed values might be attributed to the effect of discrimination against the heavier isotope of carbon by Rubisco in leaves during the C_3_ photosynthetic process [43]. It has been noted that the isotopic ratio of Glc from the kernels fed under the closed ^13^C-enriched atmosphere condition increased to be 6.58%, similar to the theoretical value. By imposing the prefixed samples to the dry wind condition, Orbitrap-based MS analysis allowed us to examine the possibility of starch degradation at osmotic adjustment even with the potential discrimination effect. Consequently, we found, at least during short-term (24 h) dry wind conditions, that there was little effect on the breakdown of starch synthesized in amyloplasts. SEM observations showed no starch granules forming digestive pits at high temperature [9], [27]. Therefore, it appears that the 24-h duration examined was too short to explain the formation of chalky rings by starch breakdown alone under dry wind conditions.

Quantitative PCR analysis provided evidence that rates of starch synthesis decreased at moderately low water potential (Fig. 4B and Table S1 in File S1). Considering that sucrose accumulation accompanying decreased starch biosynthesis has been observed under mild water stress in other plants [44], [45], there might be an interaction between the reduced starch biosynthesis and sugar accumulation in developing rice endosperm. In starch metabolism, down-regulated expression of *GBSS1*, which is responsible for amylose synthesis [46], has been observed at mild water deficit (Fig. 4B). In addition, down-regulation of *ISA1* expression (for debranching enzyme isoamylase 1, which releases soluble maltoligosaccharides from starch granules) at osmotic adjustment (Fig. 4B), allows us to exclude the effect of starch degradation. It is generally recognized that changes in amylopectin composition are involved in the process of chalky formation [10], [46]. Amylopectin composition was not analyzed in this study, although it is possible that amylopectin was structurally modified under the short-term dry wind conditions. The down-regulation of *GBSS1* and *BE* observed here, which was similarly observed at high temperature [10], [47], may have some effect on the lack of short-chain amylopectin formation under the short-term stress.

Aside from the reduced starch biosynthesis, the responses of other genes, such as plant hormone metabolism-associated and stress responsive genes, were quite variable. Consistent expression patterns for HSP- and LEA-encoding genes (Table S1 in File S1) showed additive responses to shoot water deficit and high temperature. Among small HSP-encoding genes, upregulation of *HSP16.9A* and *HSP26.7* was detected under dry wind conditions. *HSP16.9A* and *HSP26.7* have been associated with heat and water deficit (and anoxia) in rice kernels, respectively [48], and thus might play different roles in the cells. It has been reported that reactive oxygen species might be involved in chalky grain formation [49]. As the expression of genes that encode manganese superoxide dismutase and pyruvate Pi dikinase, recognized as the proteins targeted by thioredoxin [50], were eventually downregulated at 24 h (see *MSD1* and *cyPPDK* in Table S1 in File S1), dry wind stress might have disturbed the redox homeostasis and modified cellular metabolism at low water potential. In addition, expression of *ABA8ox2*, considered to be one of the key genes in modulating ABA levels [51], increased 2.2-fold in the moderately stressed kernels (Table S1 in File S1). It remains unclear whether the expression of several genes analyzed at the kernel level under dry wind conditions reflect the responses that occurred in endosperm layers that developed chalkiness. Therefore, it is necessary to perform spatial analysis of metabolic changes that occur prior to chalky ring formation in the further study–a subject we are currently investigating.

Early studies of rice endosperm showed that starch accumulation occurs from the center towards outward in the endosperms [5], [6], and it is important to keep in mind that our conclusion agrees well with the general consensus on the accumulation pattern. Also, it is reasonable to assume that cell expansion by osmotic adjustment precedes starch accumulation in the rice endosperm cells. In osmotically adjusted cells, imported sugars would be accumulating faster than consumed for growth (including wall biosynthesis and respiration), and the rest might be utilized for the potential substrate(s) of starch and fatty acids to be synthesized in plastids. The fate of sugars at the subcellular level remains unclear, however, the most likely explanation based on our findings is that sugars to be synthesized to starch kept accumulating in vacuoles and cytosol in the cells, so that the cells could be expanded by maintaining water volume in size and cell Ψ_p_ [37], [52], followed by starch accumulation. It is reasonable to assume that vacuole’s volume in the cells would be maintained during osmotic adjustment. The early microscopic observations showed that increasing the packing density of amyloplasts in the cells was closely associated with distorting of the volume occupied with other organelles including vacuole which are filled with water containing small molecules [7], [53]. The situation might have been altered when osmotic adjustment turned on. It is possible that the vacuolar volume might have preserved in cytosol without replacing their volume to amyloplasts’ volume during osmotic adjustment. If this were the case, then in addition to the reduction in starch biosynthesis, the presence of vacuoles stayed in the cytosol as a consequence of osmotic adjustment is anticipated to have an impact on the formation of air space in the cells, which would occur during the kernel dehydration.

In addition to the above-mentioned metabolic changes observed at low water potential, the size heterogeneity of amyloplasts microscopically observed in the dry wind-treated kernels was due to the presence of a number of small amyloplasts formed with the relatively less large amyloplasts (Fig. 5C). Zakaria et al. [9] similarly observed many small amyloplasts containing single starch granules that created large air spaces at high temperature, consistent with our observation. Little is known about the molecular basis for the downsizing of amyloplasts in the chalky area, but several possibilities may be raised. Since plastid volume is responsive to rapid changes in cell osmotic potential [54], [55], regulation of plastid (amyloplast) volume in certain endosperm cell layers may be influenced by dynamic changes in cellular water status. Secondly, the size heterogeneity of amyloplasts might be attributed to abnormal development, such as a failure of plastid division [56]. Third, the metabolic speed of fatty acid synthesis in plastids may be retarded under dry wind conditions. Because PEP and pyruvate in cytosol act as precursors for several pathways (including fatty acid biosynthesis and amino acid metabolism) that occur in plastids and require carbon and ATP, the 50% reduction in expression of *cyPPDK* (Table S2 in File S1), which helps to modulate carbon and lipid metabolism [57], suggests that primary metabolism might have been inhibited in the developing plastids. The physiological role of *cyPPDK* has not yet been well clarified; however, the down-regulation of *cyPPDK* detected here and similarly observed at high temperature (e.g., reference [10]) may be involved in similar metabolic deterioration under stress conditions. All of these possibilities will need to be addressed further.

In conclusion, this work has demonstrated that foehn-induced hot and dry wind conditions in ripening rice grains induce chalky ring formation within the shortest time-frame known to have been reported, resulting in deterioration of rice appearance. Combined analyses using a pre-fixed ^13^C tracer and qPCR assay indicated that partial inhibition of starch biosynthesis without significant starch degradation during osmotic adjustment was strongly associated with ring-shaped chalkiness in the growing endosperm cells. As indicated in the previous work [4], kernel growth was sustained at control levels by osmotic adjustment, with a temporary increase in sugar delivery to the kernels under dry wind conditions. Based on these results, we propose that ring-shaped chalkiness was closely associated with moderately low water status that developed at high VPD, which was increased at high temperature. Independent differences between short-term dry wind and long-term high temperature remain to be further clarified. Considering the different types of chalkiness observed in kernels, it would be useful to conduct a spatial analysis in the developing endosperm to understand the formation of rice chalkiness under environmental stress. The application of Orbitrap-based MS with the stable isotope(s), that we used as a pre-fixed ^13^C tracer in this work, together with the cellular analysis, such as cell metabolomics (e.g., Reference [58]), will shed additional light and help to provide many valuable insights into environmental plant science.

## Supporting Information

File S1 Table S1, *P* values, ratio to control, and relative expression of 39 differentially expressed genes analyzed in the developing caryopsis after 24 h dry wind treatment. Table S2, Primer sequences used in quantitative RT-PCR. Figure S1, (A) Nano-ESI mass spectrum (positive ion mode) of the ethanol-insoluble fraction from ^13^C-pre-fixed kernels after partial starch-degradation treatment (see Materials and Methods). (B) Expanded mass spectrum of the range *m/z* = 203–205 in (A). Since sodiated glucose (*m/z* = 203) was the most abundant ion among those containing glucose ([Glc + H]^+^, [Glc + Na]^+^, and [Glc + K]^+^) produced by ESI in the Exactive Orbitrap MS, the signal intensity of ions with *m/z* = 204 (C_5_^13^CH_12_O_6_+^23^Na; Mm = 204.0561693) and *m/z* = 203 (C_6_H_12_O_6_+^23^Na; Mm = 203.0527693) was used to calculate the isotopic ratio for glucose. (C) Nano-ESI mass spectrum (positive ion mode) of the buffer solution used in the experiment (blank spectrum).
(DOCX)
