# Supplementary material for: Rice Chalky Ring Formation Caused by Temporal Reduction in Starch Biosynthesis during Osmotic Adjustment under Foehn-Induced Dry Wind
Source: PLoS One. 2014 Oct 20;9(10):e110374. doi: 10.1371/journal.pone.0110374 (PMC4203794; doi:10.1371/journal.pone.0110374)
Supplement: File S1 — Table S1, P values, ratio to control, and relative expression of 39 differentially expressed genes analyzed in the developing caryopsis after 24 h dry wind treatment. Table S2, Primer sequences used in quantitative RT-PCR. Figure S1, (A) Nano-ESI mass spectrum (positive ion mode) of the ethanol-insoluble fraction from 13C-pre-fixed kernels after partial starch-degradation treatment (see Materials and Methods). (B) Expanded mass spectrum of the range m/z = 203–205 in (A). Since sodiated glucose (m/z = 203) was the most abundant ion among those containing glucose ([Glc + H]+, [Glc + Na]+, and [Glc + K]+) produced by ESI in the Exactive Orbitrap MS, the signal intensity of ions with m/z = 204 (C5 13CH12O6+23Na; Mm = 204.0561693) and m/z = 203 (C6H12O6+23Na; Mm = 203.0527693) was used to calculate the isotopic ratio for glucose. (C) Nano-ESI mass spectrum (positive ion mode) of the buffer solution used in the experiment (blank spectrum). (DOCX) [file pone.0110374.s001.docx]

| Table S1: *P* values, ratio to control, and relative expression of 39 differently expressed genes analyzed in the developing caryopsis at 24h dry wind treatment | | | | | | |  |  |
| --- | --- | --- | --- | --- | --- | --- | --- | --- |
| Metabolism | Gene name | Description | RAP-ID | Accession no. | *P-*value | Ratio to | Relative gene expression of | |
|  |  |  |  |  |  | Control = 1 | Internal control = 1 | |
|  |  |  |  |  |  |  | Control | Wind |
| Starch degradation | *Amy1A* | α-amylase 1A | Os02g0765600 | AK101744 | 0.255 | 0.81 | 0.00010 | 0.00008 |
|  | *Amy2A* | α-amylase 2A | Os06g0713800 | X64619 | 0.966 | 1.04 | 0.00287 | 0.00290 |
|  | *Amy3A* | α-amylase 3A | Os09g0457400 | AK063988 | 0.356 | 1.71 | 0.00008 | 0.00013 |
|  | *Amy3D* | α-amylase 3D | Os08g0473900 | AK073487 | 0.941 | 1.34 | 0.00026 | 0.00027 |
|  | *Amy3E* | α-amylase 3E | Os08g0473600 | AK064300 | 0.395 | 0.71 | 0.13967 | 0.09662 |
|  | *BAM3* | β-amylase 3 | Os03g0141200 | AK068968 | 0.054 | 1.37 | 0.06537 | 0.08877 |
|  | *BAM5* | β-amylase 5 | Os10g0565200 |  | 0.119 | 3.33 | 0.02026 | 0.05338 |
|  | *BAM9* | β-amylase 9 | Os03g0351300 |  | 0.259 | 0.67 | 0.13707 | 0.08609 |
| Starch biosynthesis | *AGPS1* | ADP-glucose pyrophosphorylase small subunit 1 | Os09g0298200 | AK073146 | 0.021 | 0.65 | 2.08240 | 1.35548 |
|  | *AGPS2b* | ADP-glucose pyrophosphorylase small subunit 2b | Os08g0345800 | AK103906 | 0.143 | 0.68 | 13.72433 | 9.27044 |
|  | *AGPL1* | ADP-glucose pyrophosphorylase large subunit 1 | Os05g0580000 | AK100910 | 0.046 | 1.67 | 0.78107 | 1.29442 |
|  | *AGPL2* | ADP-glucose pyrophosphorylase large subunit 2 | Os01g0633100 | AK071497 | 0.851 | 0.99 | 12.90086 | 12.45538 |
|  | *SS2a* | Starch synthase Ⅱa | Os06g0229800 | AF419099 | 0.213 | 0.84 | 1.18719 | 0.99233 |
|  | *SS3a* | Starch synthase Ⅲa | Os08g0191500 | AY100469 | 0.448 | 1.62 | 0.04521 | 0.06358 |
|  | *GBSS1* | Granule-bound starch synthase 1 | Os06g0133000 | AK070431 | 0.016 | 0.68 | 10.67947 | 7.28803 |
|  | *BE1* | Branching enzyme Ⅰ | Os06g0726400 | AK119436 | 0.652 | 0.93 | 21.28256 | 19.72678 |
|  | *BE2b* | Branching enzyme Ⅱb | Os02g0528200 | D16201 | 0.072 | 0.59 | 11.41763 | 6.73170 |
|  | *ISA1* | Isoamylase 1 | Os08g0520900 | AB015615 | 0.009 | 0.55 | 1.66010 | 0.90130 |
|  | *PUL* | Pullulanase | Os04g0164900 | D50602 | 0.224 | 0.76 | 9.26717 | 6.99700 |
| Sugar metabolism | *CIN2* | Cell wall invertase 2 | Os04g0413500 | AK072276 | 0.255 | 1.45 | 0.08928 | 0.12911 |
|  | *CIN7* | Cell wall invertase 7 | Os09g0255000 | AK069080 | 0.935 | 0.98 | 0.00446 | 0.00434 |
|  | *INV3* | Vacuole invertase 3 | Os02g0106100 | AK072245 | 0.933 | 1.04 | 0.00040 | 0.00041 |
|  | *SuSy2* | Sucrose synthase 2 | Os06g0194900 | AK072074 | 0.178 | 1.61 | 1.29483 | 2.00023 |
|  | *SuSy3* | Sucrose synthase 3 | Os07g0616800 | AK100306 | 0.763 | 1.09 | 2.51794 | 2.68117 |
|  | *SuSy4* | Sucrose synthase 4 | Os03g0340500 | AK102158 | 0.186 | 1.33 | 1.04012 | 1.37994 |
| Sugar transport | *BT1-2* | Plastidial ADP-glucose transporter | Os02g0202400 | AK107368 | 0.266 | 0.80 | 18.00376 | 14.19941 |
|  | *SUT1* | Sucrose transporter 1 | Os03g0170900 | AK100027 | 0.631 | 0.92 | 0.22195 | 0.20275 |
|  | *SUT2* | Sucrose transporter 2 | Os12g0641400 | AB091672 | 0.593 | 0.88 | 0.06576 | 0.05778 |
| Pyruvate metabolism | *cyPPDKB* | Cytosolic pyruvate orthophosphate dikinase | Os05g0405000 | D87745 | 0.008 | 0.59 | 21.31288 | 12.55410 |
| ABA biosynthesis | *NCED2* | 9-*cis*-epoxycarotenoid dioxygenase 2 | Os12g0617400 | AY838901 | 0.463 | 0.88 | 0.00629 | 0.00537 |
| ABA degradation | *ABA8ox2* | ABA 8'-hydroxylase 2 | Os08g0472800 | AK120757 | 0.024 | 2.22 | 0.11716 | 0.23939 |
| Stress response | *HSP16.9A* | Small heat shock protein 16.9A | Os01g0136100 | X60820 | 0.046 | 2.42 | 0.33873 | 0.81013 |
|  | *HSP17.9A* | Small heat shock protein 17.9A | Os03g0266300 | AK104129 | 0.092 | 1.64 | 0.59144 | 0.95997 |
|  | *HSP18.0* | Small heat shock protein 18.0 | Os01g0184200 | AK071240 | 0.163 | 2.96 | 0.09253 | 0.25675 |
|  | *HSP26.7* | Small heat shock protein 26.7 | Os03g0245800 | AK120048 | 0.003 | 6.34 | 0.00861 | 0.05351 |
|  | *LEA1* | Late embryogenesis abundant protein 1 | Os04g0589800 | AK063682 | 0.959 | 1.02 | 2.71534 | 2.73991 |
|  | *LEA8* | Late embryogenesis abundant protein 8 | Os05g0584200 | AK061818 | 0.000 | 1.36 | 0.67543 | 0.91623 |
|  | *LEA21* | Late embryogenesis abundant protein 21 | Os05g0349800 | AK063677 | 0.036 | 1.25 | 5.01245 | 6.28211 |
|  | *MSD1* | Mn-superoxide dismutase 1 | Os05g0323900 | AK070528 | 0.025 | 0.79 | 1.21264 | 0.95730 |

| Table S2. Primer sequences for quantitative RT-PCR | | |  |  |  |
| --- | --- | --- | --- | --- | --- |
| Gene name | Primer sequence (5′→3′) | | Primer location^a^ | Size (bp) | Reference |
| *ABA8ox2* | F: | CTACTGCTGATGGTGGCTGA | S | 117 | [51] |
|  | R: | CCCATGGCCTTTGCTTTATC |  |  |  |
| *ACS6* | F: | CGGGATACTTGCCTTGAAGATGGC | D | 115 |  |
|  | R: | CATTGCAGGAGTTGCACCAGATG |  |  |  |
| *AGPS1* | F: | ACGCCTTAATCCCTAGCGGAAC | D | 75 |  |
|  | R: | TGCTGCAAGGCCCAACCTTATG |  |  |  |
| *AGPS2a* | F: | ACTCCAAGAGCTCGCAGACC | D | 69 |  |
|  | R: | CAATCTAGTCCCTGCACCAC |  |  |  |
| *AGPS2b* | F: | CAACAATCGAAGCGCGAGAAAG | D | 128 |  |
|  | R: | CAATCTAGTCCCTGCACCAC |  |  |  |
| *AGPL1* | F: | CATGTGCTCCTGTTGGAGAGAGTC | D | 76 |  |
|  | R: | TGAATTACACGGCCTGAACTGTCG |  |  |  |
| *AGPL2* | F: | TACTGGAACTGCACGATGTGTG | D | 73 |  |
|  | R: | GGGAGGATTGTGTCCGAAGATGTG |  |  |  |
| *Amy1A* | F: | GATACGACGTCGAACACCTC | S | 186 | [12] |
|  | R: | CGGATCGGATACAGCTCGTTG |  |  |  |
| *Amy1C* | F: | TATCATGGAGGCTGACAGCG | S | 175 | [12] |
|  | R: | GCTAATTGTGCCTCTCCACC |  |  |  |
| *Amy2A* | F: | GCCGATCATCGCACCTCTTC | S | 149 | [12] |
|  | R: | CGATCCCACATATCAGTGACG |  |  |  |
| *Amy3A* | F: | GAGGGTCATCACCAAGATCG | S | 166 | [12] |
|  | R: | TGTGTAGCTAGCTTGCGAGC |  |  |  |
| *Amy3D* | F: | GTAGGCAGGCTCTCTAGCCT | S | 90 | [12] |
|  | R: | CCAACGGTTACAAACTGCGTGA |  |  |  |
| *Amy3E* | F: | GAAGGAAGGCCTCAGGGTTC | S | 152 | [12] |
|  | R: | GCTCGTACACATCTCGCAGCA |  |  |  |
| *BAM3* | F: | TCGGCGACTCAGTCACTATACCAC |  | 115 |  |
|  | R: | AGCGAGAGGTATTCGTAGTTGCG |  |  |  |
| *BAM5* | F: | GTAGGATTGGGGCCTTGTGGTG |  | 120 |  |
|  | R: | CAGCGACGCCCTCATGTACTTG |  |  |  |
| *BAM9* | F: | GACGAGCTCCCGGTTCTTGG |  | 134 |  |
|  | R: | CCATTGGGACCCAAGCTCAC |  |  |  |
| *BE1* | F: | TTGGTGGCCATGGAAGAGTTGG | D | 74 |  |
|  | R: | TTCTGGTACTCCTGGCATTCCC |  |  |  |
| *BE2b* | F: | TTTGGCAGGATCCATCACAC | D | 130 |  |
|  | R: | TGGTGTTCTCATTCCGCTGG |  |  |  |
| *BT1-2* | F: | AGGTTGCGTGAAGTGGTTTGGG | S | 79 |  |
|  | R: | TGCTTCCATCATCACACCCATGC |  |  |  |
| *CIN2* | F: | TTCTCAAGGACAGGGTGGTCAAGC | D | 70 |  |
|  | R: | TCAGCCTGTGCAGTTTGTAGCC |  |  |  |
| *CIN7* | F: | TGTGCACCGACTTGACAAAGTCG | D | 101 |  |
|  | R: | GCGATATGGTTTTGTGATCGTCG |  |  |  |
| *cyPPDKB* | F: | ATGGCTCCGGCTCAATGTGC | S | 85 |  |
|  | R: | GGTCCTTCATGGCCTTGTTGC |  |  |  |
| *GBSS1* | F: | TCCGTCATTCCTGGAGAAGGTTTG | D | 69 |  |
|  | R: | TCAACTCCAGTGTCAGGTCCGTAG |  |  |  |
| *HSP16.9A* | F: | ACTCCCAAATCGCCCTCTTTGC | S | 67 |  |
|  | R: | TGATGCGAAGTTGTGGGCTACTG |  |  |  |
| *HSP17.9A* | F: | TTGGCCTGGTTTGTCTGTCGTG | S | 68 |  |
|  | R: | ACGGACACACACTGGACTCAAC |  |  |  |
| *HSP18.0* | F: | AGATTCCGCATCTCTCTGCATCTG | S | 75 |  |
|  | R: | AGTCACAACTCACTCAACTCAAGC |  |  |  |
| *HSP26.7* | F: | TCAGCGAACGAGCGAATGAATGG | S | 74 |  |
|  | R: | AGGCATGAGGTGATGAAGCAACC |  |  |  |
| *INV3* | F: | TCTTGCTGGCCTGGGTTGTTTG | S | 125 |  |
|  | R: | TCCCAGGCTTTGCATCCATGTG |  |  |  |
| *ISA1* | F: | TGACTTGCAACGGTTCTGCTCTC | D | 64 |  |
|  | R: | GCCAAGGGACTCGCATTGTTTG |  |  |  |
| *LEA1* | F: | AACACGTCGCAAGTCGAAGCAC | S | 77 |  |
|  | R: | ACGTGCGTTGCGTATCAGTGTG |  |  |  |
| *LEA8* | F: | CTGGACAGCTTGTCGTTCAAGG | S | 70 |  |
|  | R: | TGGAGACGTTGACGTTGCTGTG |  |  |  |
| *LEA21* | F: | AGCCGCAGAAGACATACACACAC | S | 69 |  |
|  | R: | CGCTGCGAACTGATCGATGAAC |  |  |  |
| *MSD1* | F: | GTGGAAACAACTGCTAACCAGGAC | D | 91 |  |
|  | R: | AGTACGCATGCTCCCAGACATC |  |  |  |
| *NCED2* | F: | TGCTTGTACAGACAGCCAAGGAG | S | 62 |  |
|  | R: | TCCCTATCACTGAGGGACAAAGC |  |  |  |
| *PUL* | F: | TGGGACTTTGGTGAGGTTGCAC | D | 100 |  |
|  | R: | CGCGGATCCTATCGTTGAAACTAC |  |  |  |
| *SUT1* | F: | TGCCTCGTCCTCTTTGCGTTTC | D | 62 |  |
|  | R: | GCGAAGGGAACACTGTACAGAACG |  |  |  |
| *SUT2* | F: | ACCATATGCAATGGCTGCTAGTCG | D | 112 |  |
|  | R: | ACCCAGTGACACAATAACCTGTGG |  |  |  |
| *SuSy2* | F: | TCGGAGTTCAACCACAGGTTCC | D | 80 |  |
|  | R: | ATGGTGTCAAGCACACGCTTTG |  |  |  |
| *SuSy3* | F: | AGCTGAGTGTCCCTGAATACTTGC | D | 72 |  |
|  | R: | GCACAAAGTTGTTCTGGGTGCTTC |  |  |  |
| *SuSy4* | F: | AGTTCCGTGAACTGGCGAAGAC | D | 73 |  |
|  | R: | ATCACGTCGAGGACCTACAGTCAG |  |  |  |
| *SS2a* | F: | TCATTGACGCTCCTCTCTTCCG | D | 78 |  |
|  | R: | TCATGCGCTTCATGATTTCCTGTC |  |  |  |
| *SS3a* | F: | TGTGGGAGTATGTATCAGCAATG | S | 148 |  |
|  | R: | GCGCAAAAGAAACCATAGAAC |  |  |  |
| *eEF-1a* | F: | TTTCACTCTTGGTGTGAAGCAGAT | D | 103 | [22] |
|  | R: | GACTTCCTTCACGATTTCATCGTAA |  |  |  |
| *UBC* | F: | TCCGCAAGTTCGCTTCTTGACC | D | 72 |  |
|  | R: | TCCAGGCAAATCTCACCTGTCTTG |  |  |  |
| ^a^Location of forward (F) and reverse (R) primers on the same (S) or different (D) exon(s). | | | | |  |

Figure S1


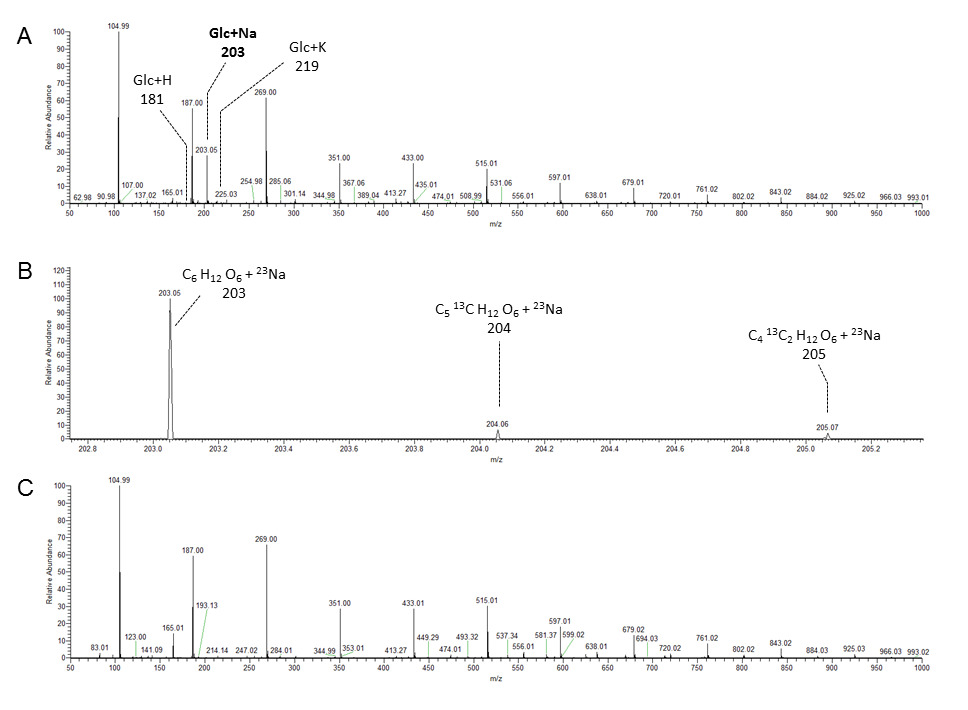


Figure S1. (A) Nano-electrospray ionization (ESI) mass spectrum (positive ion mode) of the ethanol-insoluble fraction from ^13^C-pre-fixed kernels after partial starch-degradation treatment (see Materials and Methods). (B) Expanded mass spectrum of the range *m/z* = 203–205 in (A). Since sodiated glucose (*m/z* = 203) was the most abundant ion among those containing glucose ([Glc + H]^+^, [Glc + Na]^+^, and [Glc + K]^+^) produced by ESI in the Exactive Orbitrap mass spectrometer, the signal intensity of ions with *m/z* = 204 (C_5_^13^CH_12_O_6_ + ^23^Na; Mm = 204.0561693) and *m/z* = 203 (C_6_H_12_O_6_ + ^23^Na; Mm = 203.0527693) was used to calculate the isotopic ratio for glucose. (C) Nano-ESI mass spectrum (positive ion mode) of the buffer solution used in the experiment (blank spectrum).
